# Epidemic Spread and Its Management Through Governance and Leadership Response Influencing the Arising Challenges Around COVID-19 in Pakistan—A Lesson Learnt for Low Income Countries With Limited Resource

**DOI:** 10.3389/fpubh.2020.573431

**Published:** 2020-12-10

**Authors:** Ahsan Nawaz, Xing Su, Muhammad Qasim Barkat, Sana Asghar, Ali Asad, Farwa Basit, Shahid Iqbal, Hafiz Zahoor, Syyed Adnan Raheel Shah

**Affiliations:** ^1^College of Civil Engineering and Architecture, Institute of Construction Project Management, Zhejiang University, Hangzhou, China; ^2^Key Laboratory of China Food and Drug Administration (CFDA) for Respiratory Drug Research, Department of Pharmacology, School of Medicine, Zhejiang University, Hangzhou, China; ^3^School of Life Sciences, Zhejiang University, Hangzhou, China; ^4^School of Management, Shenzhen University, Shenzhen, China; ^5^School of Agriculture and Biotechnology, Zhejiang University, Hangzhou, China; ^6^Management Studies Department, Bahria University, Islamabad, Pakistan; ^7^Construction Engineering and Management Department, National University of Sciences and Technology, Risalpur, Pakistan; ^8^Department of Civil Engineering, Pakistan Institute of Engineering and Technology, Multan, Pakistan

**Keywords:** COVID-19, preventive measures, current status, governance quick response, 169 days survey

## Abstract

The coronavirus disease (COVID-19) was first reported in China (Wuhan) at the end of 2019. It has rapidly spread over 216 countries, including the USA, UK, Europe, Russia, and many Asian countries. It has affected more than 4.5 million people, and around 0.3 million deaths have been reported globally. Many preventive measures have been adopted worldwide to mitigate its spread. The government of Pakistan has also taken many preventive measures to combat the COVID-19 outbreak, such as rapid response by governance, continuous monitoring of the pandemic spread in the affected areas, and integration of resources from multiple sectors, including health, education, defense, and media. According to global statistics, the number of COVID-19 cases in the country remained remarkably lower than the expected number for the first 169 days, as compared to other countries. A total of 286,674 confirmed cases, including 16,475 active, 6,139 deaths, and 264,060 (92%) recoveries were reported. The study finds that strict adherence to national policies, effective governance, and unity at the national level resulted in better outcomes. Hence, the preventive measures, rapid responses, and strategies adopted for combating the challenges could be adopted as a learning tool for other countries having similar work environments and financial constraints. This paper can help and guide governance/public actions in response to the possible rebound of coronavirus this fall/winter.

## Introduction

Coronavirus disease (COVID-19) is considered to be the third epidemic disease that affected more than 216 countries in the world. According to the World Health Organization (WHO), 4,589,526 cases and 310,391 deaths have been reported worldwide. To date, the highest number of positive cases are reported in the United States followed by Russia, Italy, India, the UK, and Spain (1). COVID-19 has affected the gross domestic product (GDP) of almost all countries. According to a recent study, a fall of 2% is observed in the world GDP, while the GDP of developing and industrial countries dropped by 2.5 and 1.8%, respectively. The Chinese GDP observed an overall drop of 3.7%, while their exports, imports, and households suffered a drop of 3.5, 3.2, and 7.2%, respectively. East Asia and Pacific countries are expected to have a significant loss in their trade, for example, 3.2% loss in Cambodia, 2.1% in Singapore, 2.3% in China, 3% in Thailand, and 2.7% in Vietnam (2). The most significant GDP loss of 4.6% is observed in Japan, followed by 3.4% in the United States and the European Union (3.4%), and 3.2% in Canada. Moreover, output services such as tourism declined by 8.8%, while agriculture and manufacturing industries suffered a 3% loss (3). In the same way, the GDP of Pakistan is deleteriously affected by the current epidemic spread; in the year 2019–2020, the GDP level has declined by 1.6%, and it may achieve a growth rate of only 2.9% in the year 2020–2021. It is also estimated that the currency may fall from Rs. 116.8 to Rs. 178.5 per one US dollar during 2020–2024, while unemployment may rise from 3.9 to 14.7% (4).

The travel restriction in Wuhan started on 23 January 2020, which had coercively reduced the spread of the COVID-19 outbreak by 80%, whereas travel restrictions from and to mainland China could only reduce the spread of the COVID-19 outbreak by 50%. The early screening, detection, isolation, self-quarantines, and frequent hand washing proved to be more effective preventive measures than the travel restriction (5). Shim et al. reported that the first case in South Korea was declared on 20 January 2020, but the number of cases gradually increased, as 6,284 cases with 42 mortalities were reported until 6 March 2020. It was concluded that social distancing is an effective preventive measure to slow the spread of the coronavirus outbreak (6).

The border of Taiwan and mainland China is very close, and it was thought that Taiwan would be severely affected due to frequent flight operations between them. However, using the good experiences from the 2003 SARS, the Taiwan government had effectively controlled the COVID-19 outbreak through their well-trained official teams who quickly recognized the crises, sent health messages, arranged daily briefings to the public, and strictly implemented the preventive measures, thus setting an example of how a responsible society can quickly respond to an epidemic disease (7). In Vietnam, the first case was reported on 23 January 2020, and the total number of 240 cases was reported until 4 April 2020. The government of Vietnam had implemented preventive measures such as rapid response by clear leadership, the role of media, and the provision of medical services. Therefore, no death was reported until 4 April 2020 (8).

In Nigeria, the first COVID-19 case was reported on 27 February 2020, while 318 total confirmed cases with 10 mortalities were reported on 11 April 2020. Collectively, a total of 13,814 cases with 747 deaths were reported in Africa on 11 April 2020. It was thought that COVID-19 would bring the most adverse effects in African countries compared with Europe. However, despite limited resources, the Nigerian government controlled the COVID-19 outbreak by implementing preventive measures such as area lockdown or movement restriction (9).

In Italy, there were only three cases in the first half of February 2020, but only a month later, a total of 22,512 cases with 1,625 deaths were reported on 17 March. Italy became the second most affected country after China with the highest number of cases and deaths by the time. The age of patients affected by COVID-19 is mostly 70–80 years in Italy, and 23% of the population is over 65 years old. Most of the patients who died were either of old age or had already suffered from other diseases; that is why the mortality rate was high in Italy (10). In Iran, the first death was announced on 19 February 2020, and as of 16 March, 991 cases with 853 mortalities had been reported (11). Iran is facing many difficulties regarding the ongoing COVID-19 outbreak due to the restriction imposed by the USA for the last 4 years. The lack of medical supplies, such as personal protective equipment (PPE), life-saving medicines, and laboratory equipment, scaled up the burden of COVID-19 mortalities. It was urged to the USA and UN Security Council to temporarily lift or ease Iran's restrictions (12).

The Government of Spain announced the COVID-19 emergency and completely locked down the country on 14 March. A total of 13,716 confirmed cases with 598 mortalities had been reported. Spain was the second country in Europe after Italy that was severely affected by COVID-19. A shortage of medical supplies had been reported, such as PPE, gloves, face masks, ventilators, and intensive care beds, due to the increasing number of admitted patients each day. Besides, dozens of healthcare workers were affected by corona, and the doctors gave priority to the older patients and started to provide guidance on the telephone (13).

The USA was the most adversely affected country in the world. According to the WHO, 1,966,932 confirmed cases and 85,860 deaths had been reported in the USA until 17 May 2020 (14). The total number of infected cases and deaths and the preventive measures adopted by different countries to combat the COVID-19 are summarized in Table 1.

**Table 1:** Total cases, deaths, and preventive measures adopted by different countries.

**Country**	**Total cases**	**Deaths**	**CFR**	**Preventive measures**	**Sources**
Pakistan	286,674	6,139	2.1%	Organized team under NAP; lockdowns; social distancing; hand washing with soap or sanitizer; wearing mask; emphasizing on preventive measures through media; fulfilling essential medical needs such as PPEs, masks, gloves, and ventilators; and enhancing the capacity of existing hospitals and labs.	(1, 14)
South Korea	14,714	305	2%	Social distancing was implemented as a major preventive measure by the government.	(6)
Kuwait	73,068	486	0.6%	Lockdown, social distancing, tracing the contacts of infected person, and providing awareness to public regarding COVID-19 preventive measures.	(15)
Japan	50,210	1,059	2.1%	Lockdown, closing schools and public places, and increasing testing capacity for large population.	(13)
India	2,329,638	46,091	2.0%	Banned flight operations and closed shopping malls, schools, and movie theaters.	(13)
Bangladesh	263,503	3,471	1.3%	Lockdown, ban on travel, maintaining social distancing, limited working hours, and establishing remote sanitization at public places.	(16)
Iran	331,189	18,800	5.6%	Washing hand with soap or alcohol-based compound, maintaining social distancing, closing schools and markets, and banning public traffic and gatherings.	(17)
China	89,444	4,699	5.2%	Strict lockdown, early detection and reporting of suspected patients, maintaining social distancing, improving personnel hygiene, and taking rest by COVID-19 patients.	(18)
Italy	251,237	32,215	12.8%	Banned flight operation, early identification and isolation of suspected COVID-19 patients, and established quarantine centers and hospitals for disease patients.	(19)
Spain	326,612	28,579	8.7%	Lockdown and banned local places. The government ensured to provide basic facilities to hospitals.	(13)

*CFR = Number of Death / Number of Cases × 100*.

The first COVID-19 case was reported in Pakistan on 26 February 2020, and the second case was reported by the federal health ministry on the same day (20). They both came from Iran (21). On 9 March 2020, COVID-19 cases gradually increased to 16 (22). These cases quickly jumped to 461 on 19 March 2020, with the first two confirmed deaths on 18 March 2020. On 31 March 2020, the total number of cases was counted as 2,039, with 82 recoveries and 26 deaths. Moreover, on 22 April 2020, 10,000 cases were reported with 212 deaths. Comparing the performance of Pakistan with Italy and Spain, the main difference was the early implementation of lockdown (to maintain social distancing) and closing of borders (to stop the inclusion of external potential virus carriers), which contributed to the reduction of case fatality rate (CFR).

**Figure 1:**
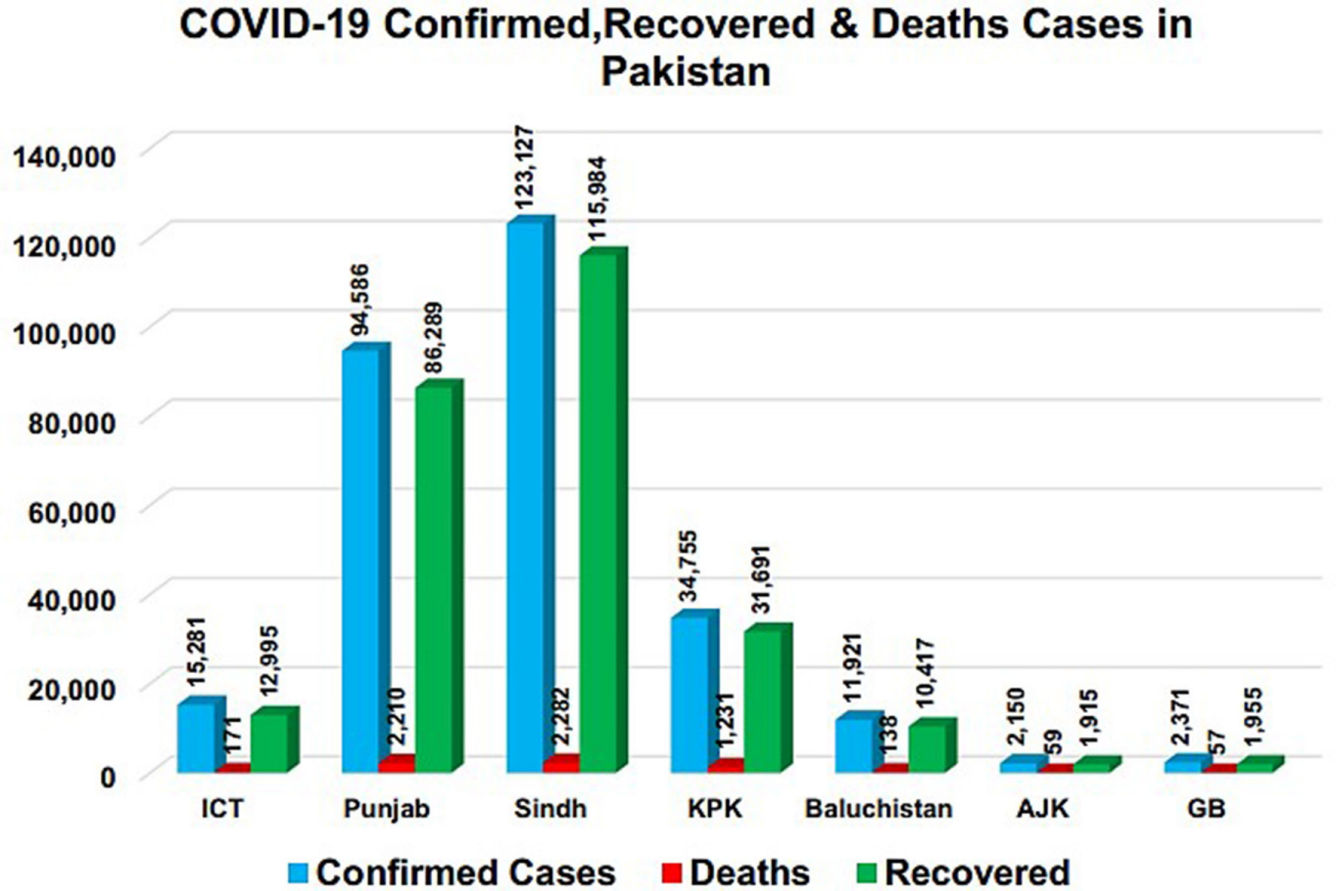
COVID-19 confirmed, recovered, and death cases.

**Figure 2:**
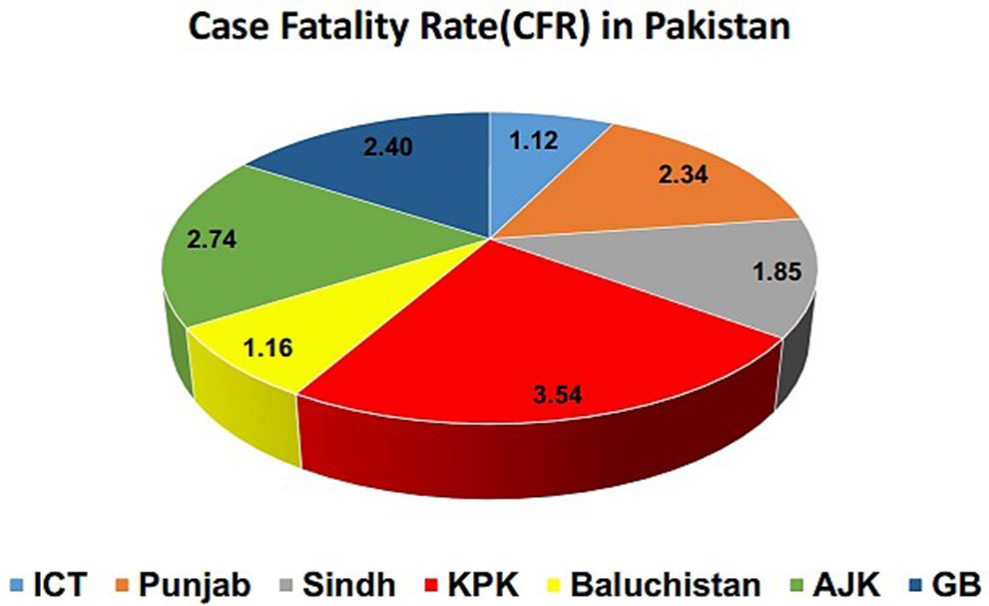
Case fatality rate (CFR).

All the confirmed cases had recently traveled from Iran, Syria, and London, which adversely affected the situation (23). On 12 August 2020, the total number of confirmed cases in the country was 286,674, with a death toll of 6,139 and 264,060 recoveries. The current scenario of COVID-19 in the country has been presented province-wise in Table 2 and Figure 1. According to this scenario, Sindh has the highest number of positive and active cases, while the highest deaths happened in Khyber Pakhtunkhwa (KPK). Likewise, a higher number of recovered cases were reported in Punjab province, and the CFR was 2.1% in the whole country. The CFR was 3.54% in KPK, 1.85% in Sindh, 2.34% in Punjab, 1.12% in Islamabad, 2.40% in Gilgit-Baltistan, 2.74% in Azad Jammu and Kashmir (AJK), and 1.16% in Baluchistan, as shown in Figure 2 (24). It was noticed that the overall recovery rate was 92% in the country, as shown in **Figure 7**.

**Table 2:** The current scenario of COVID-19 cases.

**Sr. No**.	**Province**	**Confirmed cases**	**Deaths**	**Recovered**
1	Punjab	94,865	2,179	86,389
2	Sindh	124,929	2,297	117,972
3	KPK	34,947	1,235	32,248
4	Baluchistan	12,044	138	10,458
5	Islamabad Capital Territory	15,323	173	13,020
6	Gilgit-Baltistan	2,402	58	2,002
7	Azad Jammu and Kashmir	2,164	59	1,971

*COVID-19 cases—country-based survey from 26 Feb to 12 Aug (169 days)*.

The number of COVID-19 cases is increasing gradually throughout the country; hence, there is a need to make effective strategies and chalk out the planes at the highest level to curtail the transmission. Moreover, Pakistan is a developing country having limited resources and budgets against any viral disease, particularly the highly contagious ones like COVID-19. Nevertheless, the Ministry of National Health Services, Regulation & Coordination (M/O NHSRC) introduced a National Action Plan (NAP), on 12 February 2020, for “preparedness & response for the governance, status, and situation of COVID-19 in the country.”

The central government implemented the NAP all over the country to counter the pandemic spread. The medical staff provided clinical care and health education to the public with remarkable assistance from the government. A relief package was announced for the needy and poor people. Besides, the government enforced lockdown across the country to control the spread of the pandemic. Additionally, media broadcasted daily cases and helped in disseminating critical preventive health measures through audio and video messages across the country. The National Institute of Health (NIH), National Disaster Management Authority (NDMA), and Punjab Disaster Management Authority (PDMA) were also maintaining the data of daily COVID-19 cases. As the government is endeavoring to control the pandemic spread according to WHO guidelines, this study analyzes the adopted methods/measures for their effectiveness in combating the COVID-19 pandemic spread. For evaluating the risk, the data were collected from different sources in the country.

The main objectives of the abovementioned action plan and preventive measure are to stop the spread of COVID-19 and to provide quick response challenges for governance, status, and the situation at each level (federal, provincial, and district) to make the ability for detection, prevention, and expressing responses against COVID-19 outbreak or other novel pandemic disease in the country. The epidemic COVID-19 might have a significant effect on the health status, safety, and culture of the public (25). If the government policies are strictly followed, there would be no difficulty to overtake the pandemic throughout the country.

The study was divided into four sections. The first section defines and gives an insightful picture of the origin of COVID-19 and its background. It is followed by explaining the current scenario and status of COVID-19 in developing countries, with a focus on Pakistan in the *MATERIALS AND METHODS* section. The *RESULTS* section elaborates on the results of the COVID-19 survey from 26 February to 12 August 2020 (169 days) and explains the impact of quick government response on public health and safety. The last section describes the scope of work, discussion, and conclusion.

## Materials and Methods

The following three types of data have been used in this study to analyze and predict the results.

The first type is the data collected by the WHO from 26 February to 12 August (1, 14), accessed through open sources of the WHO website. The second source of data was the local administration (district, provincial, and central level). This data was verified by the National Institute of Health (NIH), National Disaster Management Authority (NDMA), Punjab Disaster Management Authority (PDMA), and National Command and Operation Centre (NCOC). They were actively working as the leading sources to gather and eradicate the outbreak data (23, 26). NCOC contains representatives from all provinces, AJK, Gilgit-Baltistan (GB), and ICT under the supervision of the prime minister. The NDMA never had a meeting in the last 2 years that is why the NCOC was established. The third source of data was the collection of information through social media, newspapers, and electronic media. Some of the raw data was collected from the national guidelines (i.e., NAP, presentations, articles, meetings, necessary documents, and other confidential sources). The quality and criteria level were adjusted, such that the data would be collected from the national sources (NIH, Pakistan). Likewise, data collected from other sources were refined and verified through respective authorities such as the NIH, NDMA PDMA, and NCOC.

**Figure 3:**
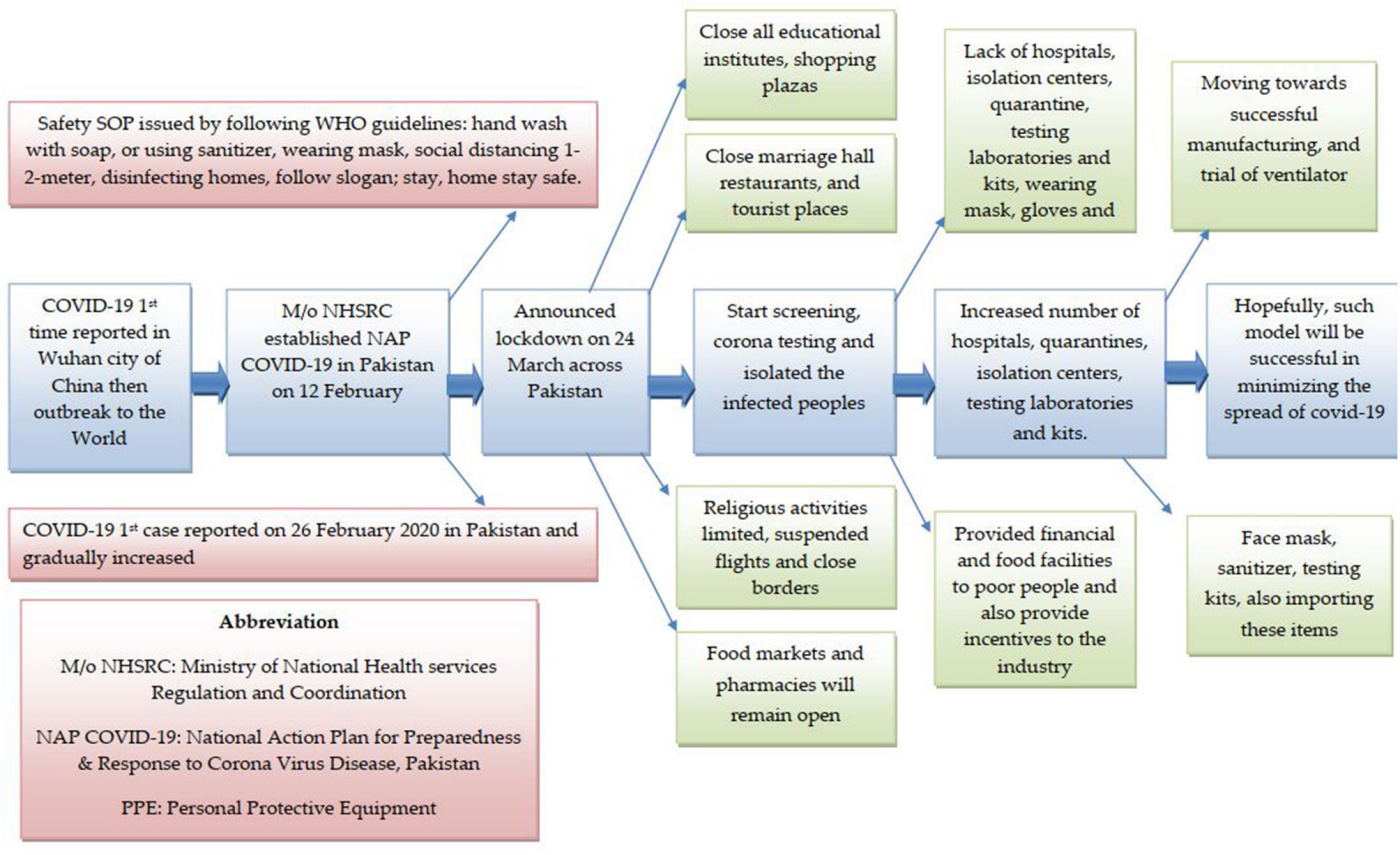
COVID-19 measures taken by the central government.

This research has reported and analyzed the data regarding the methods and policies adopted by the local and central government for quick response to mitigate the effects of COVID-19. Origin 19 and SigmaPlot 12 have been used as data analysis and graphing tools (27, 28). The study has provided a real picture of the number of identified COVID-19 cases. It has evaluated the effectiveness of the government's quick response for public health safety by mitigating the effects of pandemic spread (Figure 3). The study is narrative by its nature, and a thematic approach was adopted to analyze and identify the emerging lessons and guidelines.

## Results

The central government is endeavoring to take all immediate measures to stop the spread of COVID-19 among the masses. Since 26 February 2020, when the first COVID-19 case was reported in Karachi (Sindh), all available resources were utilized to ensure the safety of the public. Meanwhile, the travel history data of all suspected cases were collected and mitigation strategies with all services and measures against COVID-19 were shared with the public. It was followed by collecting the contacts' history for early detection of suspected cases, promoting social distancing and isolation, and establishing quarantine facilities. Figure 3 shows that many effective measures have been taken by the government against COVID-19 transmission in the country.

### Preventive Measures and Rapid Response by Leadership

The NIH arranged an awareness lecture on coronavirus for health professionals and conducted training for doctors and paramedics on infection prevention and control with a particular focus on COVID-19 (23, 29). The government has suspended the international flight operation with China from 26 January to 30 January 2020 in response to the COVID-19 outbreak. The Civil Aviation Authority (CAA) started the screening on Karachi, Islamabad, Lahore, and Peshawar airports for every passenger who came from China (30). The president advised the people in a special media conference to not participate in social gatherings, stop handshaking or hugging, and follow prescribed precautions if any COVID-19 symptoms appear (31). The government suspended all flight operations except Lahore, Islamabad, and Karachi on 13 March 2020 to stop the coronavirus spread. It was decided in the National Security meeting on 13 March 2020 to close all schools, colleges, and universities until 5 April, but it was decided again on 27 March that all academic campuses will remain closed until 31 May due to the increasing number of COVID-19 cases and the teacher will arrange online classes. The government also decided to close all land borders from 16 March and all flights will remain closed from 21 March to the end of April. The railway minister suspended 42 train operations (32, 33). The National Day, 23 March, was celebrated without public gathering and military parade. The president and prime minister requested the nation to defeat the coronavirus with unity, passion, patience, and discipline (31). The military was given orders to assist the medical facilities by using all resources in fighting the coronavirus (34). The Chinese government was also approached to assist in fighting the coronavirus. Accordingly, a team of Chinese health experts arrived in the country. The NIH welcomed the Chinese team; arranged seminars; and made effective discussion on COVID-19 about how to treat, diagnose, control, and manage COVID-19 (35). The State Bank ordered all banks to ensure that cash collected from all clinics and hospitals would be disinfected, sealed, and quarantined before making it available to the public in the market (36).

### Provision of Medical Services and Emergency Public Health Response

The NDMA chairman stated that the government was working on making personal protective equipment (PPE) and other resources for healthcare staff. The Health Ministry briefed that 14 metric tons PPE, thermometers, facemasks, gloves, and gowns had been dispatched by the federal government to provinces (33). It was announced that if any person or medical staff dies during the treatment of COVID-19, he/she would be considered as a martyr, and a health relief package would be given to the bereaved family (24, 32, 33). A guard of honor was presented to doctors and paramedic staff as a mark of respect by the army and police on 27–29 March, because they are fighting as frontline soldiers against COVID-19 across the country. The citizens together with celebrities raised white flags from their roofs and balconies on 27 March across the country to express their love and passion for healthcare staff who are selflessly fighting against COVID-19 (37). The federal government also decided to provide a 1-month additional salary package to healthcare staff regarding their services against COVID-19 (38). Moreover, the NIH is playing an important role in the COVID-19 outbreak. Before the COVID-19 outbreak in the country, the NIH had already raised radical steps to stop the COVID-19 disease transmission. On 22 January 2020, the NIH arranged an awareness lecture about COVID-19 disease for healthcare professionals on a priority basis. On 10 February 2020, there was a training session on strengthening chemical and biological waste management, while on 18 February 2020, the NIH conducted a training on infection prevention and control for doctors and paramedics. On 26 February, the Health Ministry introduced NAP for preparedness and response for COVID-19. According to this plan, standard operating procedures (SOP) and guidelines are given to control and prevent the COVID-19 through epidemiological action such as how to early detect, observe, and isolate the suspected and confirmed COVID-19 cases. Such epidemiological action became compulsory for everyone who entered the country from 26 February onwards, once the first case was reposted in the country. A helpline 1166 was established for reporting COVID-19 cases across the country. Total confirmed and active cases as well as death and recovered cases were broadcasted daily on every moment by almost all national channels. The designated hospitals, isolation and quarantine centers, and testing laboratories for COVID-19 are established in the country in collaboration with the NIH. The frontline soldiers such as healthcare staff played a critical role against COVID-19. They facilitated the public by providing health education and preventive services. They helped in identifying and separating the people who had come recently from the epidemic areas or remained in contact with COVID-19 patients and guided them through NIH guidelines. They also identified the local isolation centers, provided physical monitoring such as fever, and referred the suspected cases to designated places if necessary (24, 26).

### Designated Hospitals for COVID-19 in the Country

**Figure 4:**
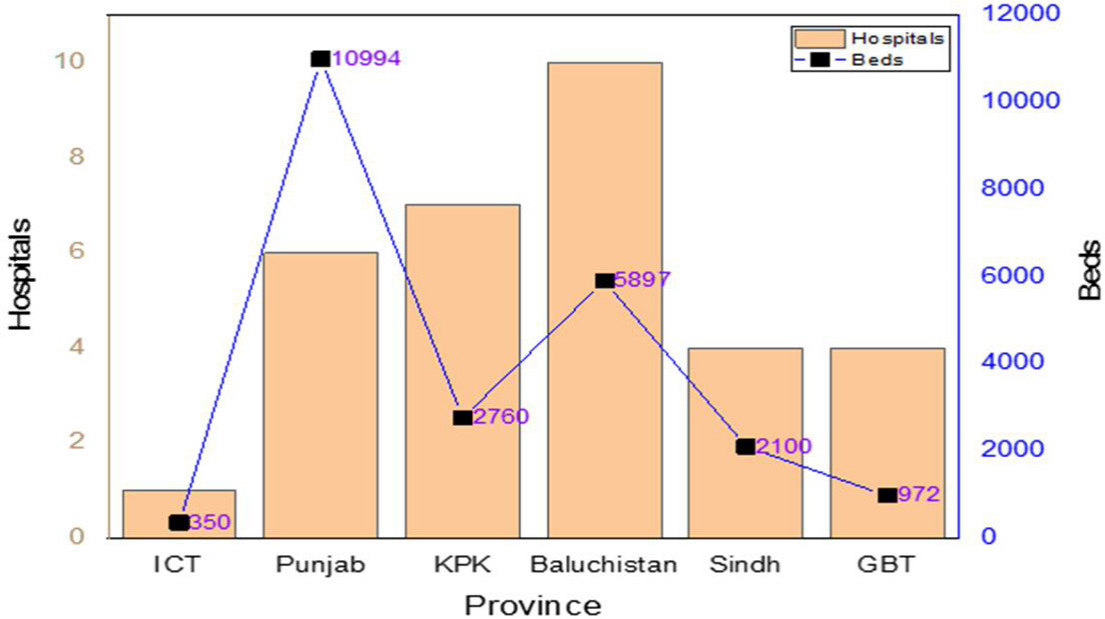
Designated hospitals to treat COVID-19 patients.

The government has implemented many policies and preventive measures for improving the governance system, situation, and status of the COVID-19 outbreak in the country. Many hospitals are fighting against COVID-19 for public health. Moreover, the government has approved specific hospitals for suspected and confirmed COVID-19 patients. Such hospitals are equipped with PPE, diagnostic laboratory, and other necessary equipment. The nominated focal person of infection prevention and control (IPC) is responsible to make sure that the IPC guidelines are followed. The guideline/SOP for waste management at airports, hospitals, and other places has been implemented; likewise, SOP was also introduced for disinfection and environmental decontamination. In Islamabad, there is only one hospital with 350 beds, while Baluchistan has 10 hospitals with 5,897 beds, Khyber Pakhtunkhwa (KPK) has seven hospitals with 2,760 beds, Punjab has six hospitals with 10,948 beds, Sindh has four hospitals with 2,100 beds, Gilgit-Baltistan has four hospitals with 972 beds, and Azad Jammu and Kashmir (AJK) has three hospitals with 530 beds as shown in Figure 4 (39).

### Province-Wise Hospitals With Isolation Facilities for COVID-19

To mitigate the transmission of infections, infected patients are separated from other patients. The designated hospitals for isolation were distributed district wise. Only one isolation center is available in Islamabad containing 10 beds. Baluchistan has 11 districts and 14 medical services with 534 beds, whereas 33 districts and 110 medical facilities are available with 856 beds in KPK. Punjab has 34 districts, and 50 medical facilities are functional with 955 beds. Sindh has four districts, and four medical facilities are functional with 151 beds. Gilgit-Baltistan (GB) has 10 districts and 21 medical units with 126 beds, while the Azad Jammu and Kashmir have nine districts and 15 medical units with 310 beds. The number of patients and beds depends on the active cases of COVID-19 (24, 39).

### Province-Wise Quarantine Facilities for COVID-19

Quarantine was implemented to separate or restrict routine activities of suspected patients in non-medical care unit. The symptoms of such suspected patients have not been exposed yet but might get exposed after some days. The primary objective is to monitor the symptoms and early detection of contagious disease such as coronavirus. The total number of quarantine centers are 23,557 in 139 districts throughout the country. Islamabad holds two quarantine centers with 350 beds. Sindh has two with 2,100 beds, Azad Jammu and Kashmir has four with 530 beds, Punjab has six with 10,948 beds, Baluchistan has 10 with 5,897 beds, KPK has 52 with 5,760 beds, while Gilgit-Baltistan has 63 with 972 beds (23, 39).

### Testing Facilities in the Country

The polymerase chain reaction (PCR) is being used globally as a reliable method for COVID-19 detection. The government implemented the PCR protocol. There were 15 laboratories since the COVID-19 outbreak started. To expand the testing capacity on a daily basis, the NDMA is collaborating with the NIH to increase the number of laboratories. Now, 57 laboratories are working throughout the country, such as Islamabad-04, Baluchistan-03, KPK-06, Punjab-18, Sindh-09, AJK-03, Gilgit-Baltistan-02, and Armed Forces-12. These laboratories are equipped with a PCR system, providing free test facilities for the public for COVID-19 detection. Such laboratories are doing 30,000 tests per day. The government has increased testing capacity from 30,000 to 280,000 and probably would be further enhanced to 900,000. A training program for medical staff was arranged, as well as the NDMA would increase the number of technicians and experts in a molecular biology lab. Some of the updated statistics about the tests are shown in Table 3 (23).

**Table 3:** Province-wise COVID-19 lab and hospitalized records.

**Province**	**Lab Status**		**Hospitalized**				**Isolation**	**Recovered**	**Deaths**
	**Tested**	**Positive**	**Stable**	**On low-flow oxygen**	**On high-flow oxygen**	**On vent**.			
AJK	29,049	2,150	11	04	01	00	161	1,915	59
Baluchistan	64,716	11,921	00	01	06	00	1,359	10,417	138
GB	16,909	2,371	70	19	01	00	269	1,955	57
Islamabad	207,700	15,281	15	20	02	22	2,056	12,995	171
KPK	223,922	34,755	34	29	65	16	1,689	31,691	1,231
Punjab	788,860	94,586	299	114	166	72	5,472	86,389	2,174
Sindh	834,655	124,127	156	69	137	36	5,463	115,984	2,282

### Impact of Lockdown/Smart Lockdown

**Figure 5:**
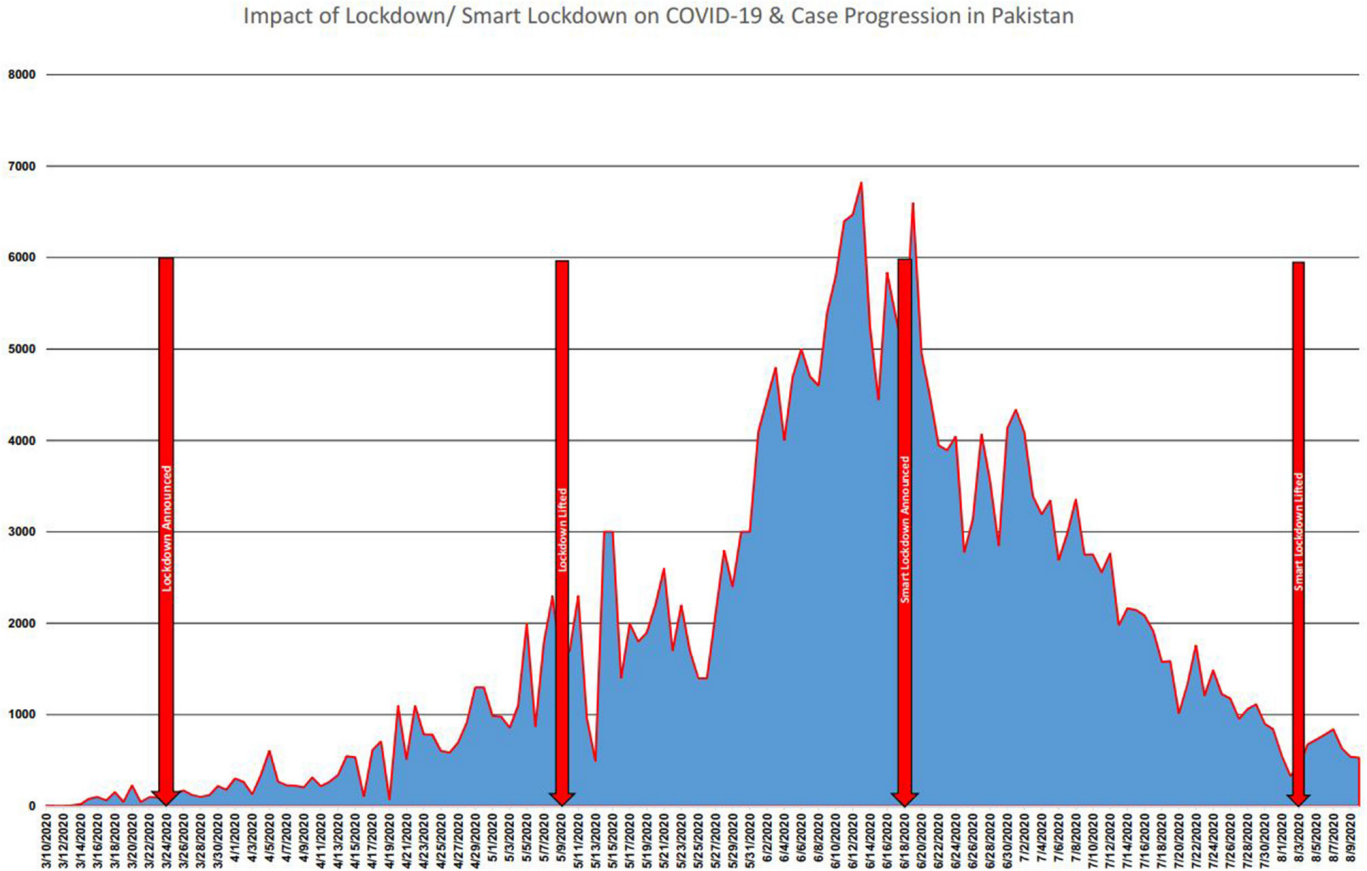
Impact of lockdown/smart lockdown on COVID-19 and case progression in Pakistan.

The lockdown started on 24 March 2020 throughout the country (32, 33, 40). On 2 April, it was further extended until 30 April. However, on 24 April, the government again decided to continue the lockdown until 9 May (30, 32, 41). After 9 May, the lockdown was lifted, but all shopping malls were closed except medicine stores; pharmacy; supermarkets; and vegetable and fruit, meat, and chicken shops. Permission was granted to only one family member to buy food items. On 18 June, smart lockdown was announced with the instructions to keep the markets open on Saturday and Sunday, while remaining closed during other weekdays. It was lifted on 3 August (Figure 5). During the period, the number of cases decreased very quickly, and the economy started to rise again. Social distancing was compulsory to anywhere, and the use of hand sanitizer and wearing a mask were made essential (37).

### Public Relief Package and Financial Assistance

The federal government issued $10 million from the World Bank to the Sindh province, and Sindh Governor Imran Ismail appreciated these steps and stated that the federal government played the central role in fighting the coronavirus and also provided the ration (food) to the families of COVID-19 patients (23, 24). The government decided on 26 March 2020 to get $3.7 billion supplementary funding from three mutual creditors and additional finance of $1.4 billion from the International Monetary Fund (IMF) to manage the COVID-19 outbreak. Besides, the country will get the extension for the $1 billion and $1.25 billion loans from the World and the Asian Development Bank. On 27 March, the prime minister announced the force designated as the Corona Relief Tiger Force (CRTF) to help the government across the cities impede the COVID-19 outbreak. This tiger force's primary objective is to supply food items sharply in a more secure or arranged way to the needy and poor people affected by COVID-19 (37, 42). The prime minister also announced Rs. 1.2 trillion relief packages on 24 March. Rs. 150 billion was allotted to laborers or low-income groups while Rs. 280 billion for wheat procurement (43). A package of Rs. 100 billion was announced to support small industries and agriculture. A Rs. 5.2-million package was fixed for the Benazir Income Support Program (BISP), and the monthly stipend of BISP has also been increased from Rs. 2,000 to Rs. 3,000 under this program. It was decided that the needy people would be selected based on local administration authority data (44). The economic relief package was approved by the Federal Cabinet on 31 March and a supplementary Rs. 100 billion emergency relief fund to mitigate the COVID-19 outbreak was issued. Another special relief package was released under the Ehsaas Program for the 12 million poor families, where cash assistance was provided to every needy person under the Kafalat Program (24). The government fund was distributed after biometric verification through the banks, as a one-time allowance for 4 months (Rs. 12,000) or in two installments of Rs. 6,000. By mid-April 2020, 1.77 million poor people received Rs. 22.466 billion as a mark of assistance (37).

### Multi-Sectoral Approach

The national media acted as a powerful weapon to educate the public and reduce hopelessness, depression, and anxiety during the COVID-19 outbreak. From the 1st day of the coronavirus outbreak, the media houses supported the NIH in disseminating the information to control COVID-19. The NIH and government's website, official newspapers, and open TV channels aired the NIH health message to the public for the eradication of COVID-19. Also, daily COVID-19 cases across the country and globally were updated. Media was allowed to trace high-risk groups of COVID-19 patients across the country and broadcast their epidemiological information at the end of March 2020, when the number of COVID-19 cases increased gradually, as many people started returning to the country. The national media invited many doctors and health experts to guide the people regarding COVID-19 diseases on daily bases. They arranged many talk shows and informative programs in which they discussed and corrected the misinformation regarding the COVID-19 outbreak. They also launched 24/7 COVID-19 hotline numbers for providing help and for reporting COVID-19 cases in case of emergency. Besides, the Aga Khan University Hospital launched a mobile app “Corona Check” for the public where they can easily monitor their symptoms and get awareness regarding COVID-19 (45). The repeated promotion of social campaigns in video messages (30 s to 1 min), such as frequent washing of hands with soap, use of hand sanitizer, wearing a face mask, and social distancing on social and electronic media, proved very fruitful in changing the public behavior toward COVID-19 pandemic. Transparent COVID-19 updates are a clear representation of government policies against COVID-19 disease. Additionally, the NIH and NDMA produced a social media account where messages are delivered to everyone on their mobile phones to give notice of COVID-19 preventive measures (25, 37).

### Impact on Country

The COVID-19 pandemic has affected the country's economy suffering a loss of Rs. 2.5 trillion (46). Yet, the government hired 60,000 people to maintain the “Plant Restoration Program,” thus reducing the unemployment rate across the country during the pandemic crisis (23). Health Minister Dr. Zafar Mirza has also requested India to lift the lockdown in Indian-occupied Kashmir and allow them to take preventive measures during the video conference of the South Asian Association for Regional Cooperation (SAARC) (30). Prime Minister Imran Khan requested the USA president to lift or ease the restriction on Iran until the COVID-19 outbreak is ended (43). The Cricket Board has postponed the Super League (47). On 16 March, it was announced that the Football League has been postponed (33, 37). Tablighi Jamaat conducted a religious gathering that has potentially stimulated the pandemic crisis in public (48). Cardinal Joseph Coutts of the Catholic Church urged his followers to strictly act upon preventive measures to reduce the transmission of coronavirus and emphasized for interfaith unity during the pandemic period. He also stated that there is no need to come to church, mosque, or other religious places for worship and requested to pray at home to save the public from the current pandemic disease (49).

The Aga Khan University (AKU) has conducted a seroprevalence survey on COVID-19 pandemic in different parts of Karachi with a low and high transmission rate in April and June. About 2,000 participants have participated in the current survey. The faculty of AKU investigated and found that 95% of people positive for COVID-19 in their blood tests were without reported symptoms such as fever, sore throat, and cough and designated as asymptomatic. According to the researcher, the ratio of asymptomatic cases in Pakistan is much more than the developed countries. The outcomes of COVID-19 seroprevalence survey showed that the pandemic virus equally spreads to adults, adolescence, and children or to men and women. Moreover, COVID-19 cases sharply rose during April and June from 0.2 to 8.7% in low transmission sites such as the Ibrahim Hyderi area, whereas it rose from 0.4 to 15.1% in high community transmission sites such as Dalmia/Shanti Nagar, Faisal Cantonment, Pehlwan Goth, and Safoora Goth. According to the federal government seroprevalence study, overall, 11% of people had been infected with the disease in Pakistan. The sharp increase in the antibody level in areas with low reported cases indicates free and continuous transmission of the virus in such a community where antibody testing rates are sub-optimal. Seroprevalence survey of antibody testing represents a clear image of the COVID-19 pandemic as they investigated asymptomatic patients that are designated as silent virus carriers of the disease. Hence, there is a need to establish effective and preventive measures to control and break the chains of COVID-19 transmission across the country (50).

### Challenge

Despite the preliminary achievement in curtailing the transmission of COVID-19 disease throughout the country, many challenges are yet to be tackled with limited resources. There is a shortage of PPE, facemasks, gloves, and medical equipment in hospitals (24, 32). The total number of designated hospitals, isolation wards in hospitals, quarantine centers, number of testing laboratories, and testing kits are not enough to cater to the public needs, due to the decline in the country's GDP and limited funds. The government is trying to look at the best suppliers from different sources to ensure sufficient medical staff according to the needs of hospitals and also increasing the number of hospitals and testing laboratories by releasing more funds. Alarmingly, the government has no exact data about those people who recently entered the country and they might be risking the whole country. Moreover, people are not well-educated; lack of awareness exists about the COVID-19 pandemic, and some people are spreading fake news. The biggest problem in the country is economic constraints. Although the government is trying to fulfill the needs of the public according to limited resources, people get panic about their food and business during the lockdown scenario. Some people are getting infected with the coronavirus across the country because they ignore the preventive measures mentioned by the NIH.

### Governance Success on Public Safety and Effective Management

**Figure 6:**
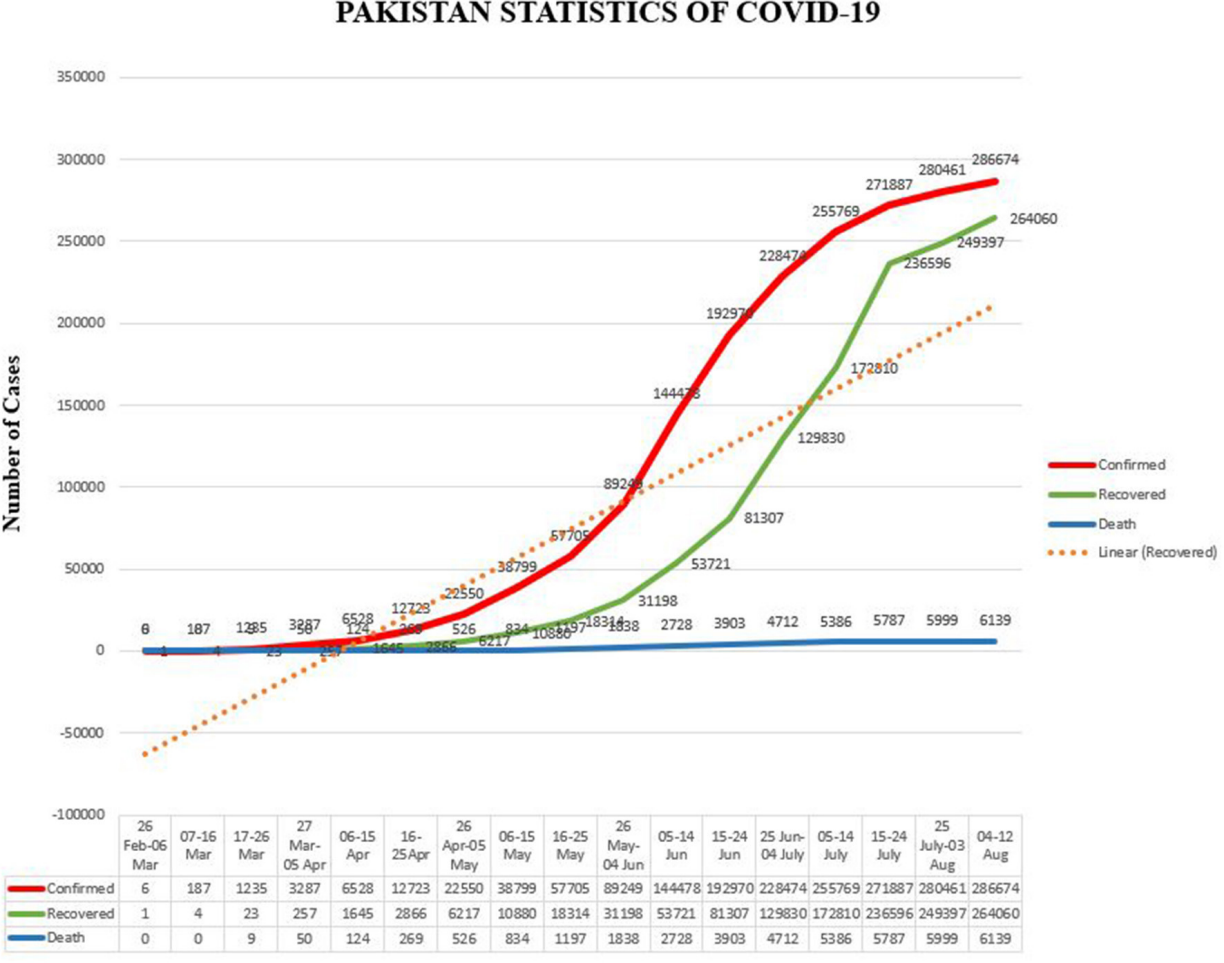
Pakistan statistics of COVID-19.

It was expected that COVID-19 would adversely affect the public health and economy of the country. Hence, the government implemented many governance policies along with imposing complete lockdown and taking preventive measures. However, the number of COVID-19 cases increased rapidly in May 2020. Being a developing country, Pakistan could not bear a complete lockdown for a long time, so Prime Minister Imran Khan decided to change it into a smart lockdown. The Bill Gates and Nobel Prize-winning scientist Michael Levitt appreciated the smart lockdown policy of the prime minister and stated that complete lockdown is not good for people's health (23). The WHO praised Pakistan's policies against the coronavirus and the head of WHO was happy to see the reduction in the number of COVID-19 cases since the end of June (14). Seven major hospitals in Lahore were cleared from COVID-19 patients, and 75–80% of COVID-19-infected patients recovered. A reduction of 28% was observed in the use of oxygen cylinders and ventilators by COVID-19 patients. Moreover, smart lockdown, coupled with an anti-corona campaign and home isolation policy for infected people, resulted in a drop of <3,000 active COVID-19 cases across the country (23, 51). A few weeks ago, the daily COVID-19 infection ratio was between 22 and 23%, but this ratio has declined to 12 and 13% per 100 patients currently, as shown in Figure 6. Due to the implementation of smart lockdown, SOPs, governance policies, and preventive measures, the number of newly reported cases of COVID-19 and deaths significantly reduced. Likewise, the number of recoveries increased after 10 July. Thus, government strategies, such as organized task force, complete lockdown, public relief funds, efficient role of health experts, social distancing, using hand sanitizer or soap to disinfect hands, wearing face mask, and especially imposing the smart lockdown, has successfully mitigated the COVID-19 outbreak. Pakistan has almost countered the COVID-19 spread, and the number of reported cases has declined up to minimum.

**Figure 7:**
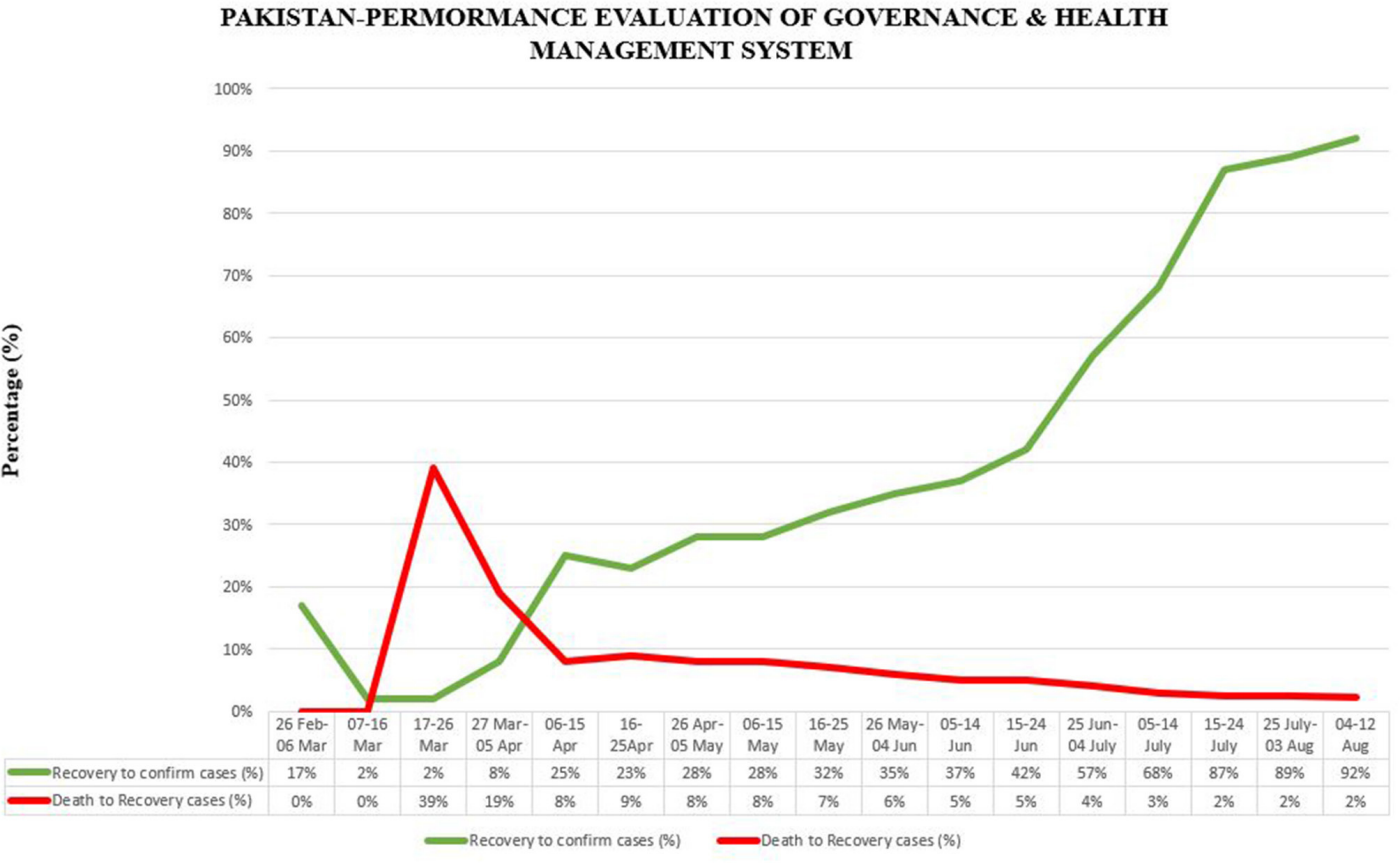
Pakistan performance evaluation of governance and health management system.

Further, a clear impact of effective government responses (health management system) and the implementation of an efficient emergency system can be observed, as the ratio of confirmed and reported cases started to reduce, as displayed in Figure 6. Another major concern was to reduce the conversion of confirmed cases to deaths, which also started to reduce after implementing an efficient management system. It can be observed in Figure 7 where green lines show improvement in patients and red lines, trending downside, show a decrease in the number of deaths.

During the COVID-19 outbreak, Pakistan has successfully overcome the COVID-19 pandemic with limited resources as compared to other developed countries. Dr. Zafar Mirza has shared his experiences and designed some strategies to control the pandemic disaster during an interview with the News Channel. He has performed his duties as special assistant to the prime minister of Pakistan for the National Health Services, Regulation and Coordination Program from April 2019 to July 2020. He has organized some rules for the improvement of COVID-19 mitigation or future pandemic disaster (52).

(a) There is no legal basis to declaring a health emergency, yet. Although such a draft bill has already been prepared and sent to the cabinet, there is a need to peruse it for future challenges.
(b) There should be a strong collaboration between government, armed forces, and civilian department, but such collaboration is too difficult between provincial and federal government for the sake of rehabilitation at each level.
(c) The NDMA was established before NCOC, but recently, the NCOC has played an effective role during the COVID-19 pandemic and needs improvements for future challenges.
(d) According to the evaluation of the International Health Regulation (2005) and Joint External Evaluation by WHO (2016), there were reforms and comprehensive recommendations initialized regarding health threats. Now, there is a need to implement the reforms and recommendations.
(e) During the COVID-19 pandemic, the Central Health Establishment has organized quarantine and screening centers at airports (19 points of entry) across the country to control the spread of coronavirus, but there is a need for more strict measures for future challenges.
(f) There is a lack of digital national disease surveillance systems and epidemiologists. It is necessary to have epidemiologists and digital disease surveillance system at each district level to control the spread of COVID-19 and any other pandemic disaster. Moreover, epidemiologists should be in the driving seat for the control of pandemic disaster.
(g) The NCOC has been transparent in updating information among people during the pandemic period, but information sharing of detailed case data is lacking, which should be improved in the future.
(h) There was a shortage of critical care experts and ICU beds (<50), but the NCOC has maintained 2,608 ICU beds across the country until 31st July. There was also an organized training program in critical care but it needs more ramp-up planning for future challenges.
(i) The houses of COVID-19 patients were marked for testing. Some people have discriminated the patients, so there is a need to change people's perception regarding diseased people.
(j) Taken together, electronic media has sent text messages to and changed the COVID-19 ring tone of 167 million mobile phone users for awareness, but some people have shared wrong information on social and electronic media. There is a need to establish strict rules for such actions.

### Immunity Level

Pakistan is located in the Asian Continent where sunlight is easily available to the public throughout the year and the duration of the winter season is very short. It is believed that the winter season is the most favorable for the transmission of epidemic diseases, such as pneumonia, typhoid fever, dengue fever, and other respiratory diseases. A hypothesis exists about sunlight and its effects that exposure to sunlight produces melatonin pigment and vitamin D. Similarly, vitamin D reduces acute respiratory tract infection (53). However, this medical stance of sunlight exposure and serum Vit-D levels of Pakistani people is still to be investigated by medical researchers. Deficiency of vitamin D causes many problems like bone problem and compromised immune system. We should maintain the optimum level of vitamin D with the required daily dose in case of deficiency because vitamin D acts as an immune modulator (46, 54). When seasonal factors change according to weather periods or region conditions, new levels of susceptibility appear among the population. Consequently, recurrent episodes of epidemic diseases may be produced each year at the same time like the current epidemic disease (55).

COVID-19 might be transmitting easily in the winter season than the summer season with an unknown mechanism like pandemic flu. It is believed that novel human coronavirus mostly attacks an immune-compromised patient, and it can still be transmitted outside of the winter period. Previously, it was believed that coronavirus does not affect children, but recent studies proved that it might be affecting children like adults (56). The optimum level of vitamin D acts as a defense against infection and can reduce viral load. Currently, there is no available standard treatment for COVID-19, but we can take vitamin D as a prophylactic measure to reduce the respiratory tract viral infection. Thus, there is a direct relationship between vitamin D and COVID-19 susceptibility and this could be used as an important element against the coronavirus pandemic (57, 58). Particularly, vitamin D is only a single source for immunity, but many other sources can provide immunity against any disease such as vitamins A, B, and C-containing plant; natural herbs; foods; magnesium; zinc; water; and micronutrients (59).

## Discussion

The COVID-19 pandemic has significantly affected the global health structure and become a colossal challenge to the world. The government of every country needs to implement effective measures and strengthen the policies for the governance system to control the COVID-19 cases in the region. The experience of dealing with COVID-19 cases necessitates implementing effective measures and strategies, not only for slowing down the spread of the COVID-19 outbreak but also for the early diagnosis, detection, prevention, and treatment SOP/guidelines. In preparation for the rapid spread of coronavirus throughout the world, the Government of Pakistan introduced many guidelines and SOPs to objectively reduce the COVID-19 transmission across the country. A task force has been established to help the public, with the strong support of law enforcement agencies, health experts, administrative staff, and policymakers. They are leaving no stone unturned to implement government policies, enforce preventive measures all over the provinces, strengthen their ability for early screening and detection of COVID-19 suspected persons, and provide timely response against COVID-19 and other pathogenic diseases. These strategies would help in eradicating and reducing the COVID-19 infection in the country. They could also be adopted as an effective prevention and control framework by other infected states who are experiencing a similar epidemic situation. The Ministry of Health has presented the NAP for Preparedness and Response to control the COVID-19. Many effective and preventive measures have been taken under this plan through collaboration with strong leadership. Keeping aside political gains, the leadership played a critical role in the implementation of preventive measures across the country at every level. During the forced lockdown throughout the country, all types of traffic and flight movements were frozen. Collective religious gatherings and social activities were banned, and even the National Day celebration (i.e., 23rd March parade, was canceled). All markets, shopping malls, mosques, and educational institutions were closed. Such strict actions were taken in the larger interest of the nation because there is only one proven treatment for such pandemic disease that is self-care and social distancing, which was rigorously enforced by the governments of South Korea, Japan, India, Bangladesh, Kuwait, Nigeria, Vietnam (1, 6, 13, 15–19), and other developing and non-developing countries.

The health system (NIH network, doctors, paramedics, and hospitals) played an important and effective role to control, prevent, and manage the spread of COVID-19. The health system provided medical facilities, courage, and patience to the whole nation as well as clear action in early diagnosing and detection of confirmed or suspected COVID-19 cases. Moreover, the isolation and quarantine SOPs were implemented strictly at each level to ensure a 14-days isolation period particularly for those who have travel histories. For morale boosting, the government decided to present tributes to the medical staff from 27 to 29 March, and the guard of honor was presented to the doctors and paramedics staff for their selfless services. Besides, white flags were raised on the roof of houses to express the love for medical practitioners who are working as frontline soldiers against the COVID-19 pandemic spread. The media also played a very supportive role in controlling the transmission of coronavirus. The social or electronic media broadcasted the repeated promotion of social campaigns in video messages (30 s to 1 min), such as frequently washing hands with soap and using hand sanitizer, wearing a facemask, maintaining social distancing, and stay home stay safe. It proved to be very fruitful in changing the public perception toward COVID-19 transmission. Likewise, transparent and quick COVID-19 updates helped to present the clear image of government policies against COVID-19 disease. During the current epidemic spread, many developing countries are facing the major problem of the decline in their GDP rate, due to closed industries and businesses and lack of import/export. However, the current pandemic could not adversely affect Pakistan's economy due to the control of the spread of COVID-19, compared with other developing countries, as summarized in Table 1. Moreover, the AKU researchers have conducted a seroprevalence survey on the COVID-19 disaster on Sep 7, 2020. In this survey, AKU collaborated with the federal government and collected data when COVID-19 cases were rising in April and June, but Pakistan has successfully overcome the COVID-19 cases in July with the implementation of strict measures taken by the government (50). Recently, Dr. Zafar Mirza has shared his experience on Sep 19, 2020, in an interview, and pointed out the lack of strategies implemented during the COVID-19 pandemic. He emphasized that we should learn from the COVID-19 disaster and improve on the things that are lacking right now in future challenges. He has identified several facts which need to be addressed for the safe future of Pakistan. These facts include the lack of a legal basis to declare a medical emergency, missing vertical collaboration between federal and provincial governments, failure of the NDMA (they never had a meeting in the last 2 years that is why the NCOC was established), lack of core capacities to respond to health threats as per IHR regulations, Central Health Establishment (CHE)'s limited power to screen people from entry points in the country, the need for a robust and real-time digital national disease surveillance system and a resourceful rapid response team for data collection and management (to monitor significance of data transparency), shortage of ICU beds and critical care specialists, and the need for proper risk communication and community engagement via direct leadership interaction and media campaigns. By addressing these factors, many developing countries can improve their health infrastructure to respond to these types of pandemic (52).

COVID-19 mostly affects immune-compromised weak patients. The immune system can be boosted up by a continuous supply of vitamin D thereby eradicating respiratory tract infection (60). Vitamin D can be obtained at an excess rate under the sunlight. Recently, a fewer number of COVID-19 cases are observed in Pakistan than other countries (23). Hence, it was thought that the decreased number of reported COVID-19 cases in Pakistan might be due to the optimum level of vitamin D that strengthens the immune system and produces protection against COVID-19. Many factors have reduced the number of COVID-19 cases, but immunity might be one factor that can help in a pandemic disaster. Moreover, vitamin D is not only the single source that can provide immunity but there are also other sources such as green leafy vegetables; vitamin A, C, and D-containing foods; water; magnesium; and zinc; they all strengthen the immune system against infection, disease, or specifically the COVID-19 pandemic (58). The central management was established to develop trust between the public and the government. The government and health ministry strongly emphasized on the central governance and leadership to fight against COVID-19. It is of note that daily health reporting data (personal and family) should be kept transparent; otherwise, trust relationships between the government and public will fade away. The central and provincial governments have enforced rigorous policies and the above-stated practices to control the on-going COVID-19 outbreak, thus resulting in a limited number of COVID-19 cases and a few deaths, as compared to China and other developed and developing countries.

## Conclusion

COVID-19 has rapidly spread worldwide in 216 countries including the USA, Spain, France, UK, Italy, and many developing countries. In this paper, on-going experiences and the current status and situation of COVID-19 cases in Pakistan are presented. Moreover, a well-tested method has been discussed to reduce the spread of the COVID-19 outbreak, which is expected to be implemented in other countries having similar financial constraints. The country's response to COVID-19 is characterized by enforcing preventive actions, rapid response by leadership, strict and smart lockdown scenarios, adequate provision of medical services and emergency public health response, as well as a multi-sectoral approach. The current situation in Pakistan regarding the COVID-19 outbreak is much satisfactory, and it needs more effectiveness in the government's performance to eradicate the impact of the outbreak. Being a developing country, financial constraints particularly falling down GDP have impeded the process of combating COVID-19, like other countries. There is a dire requirement of building new hospitals and establishing isolation centers and testing laboratories. As Pakistan successfully dealt with the current epidemic spread, despite being a developing country and having limited resources, the stated practices, policies, and experiences may be considered by other countries as “effective prevention strategies” to stop COVID-19 transmission. There is also a need to enhance public awareness to follow the preventive measures, maintain social distancing, stay at home, and not go outside without any emergency. If the public positively collaborates with the government, task force, and law enforcement agencies, the war against COVID-19 can easily be won. The government is endeavoring to develop a COVID-19 vaccine in collaboration with Sinopharm (a Chinese pharmaceutical company) since April 2020 (23). It is believed to be the best medical innovation project in the research history of Pakistan. The findings of this study will help the key stakeholders in the government to improve the health management system in the country during the fight toward COVID-19 eradication and also in preparation for the potential coronavirus rebound in the coming fall/winter. It also could be used as a tool for combating coronavirus in other countries having equal working performance and similar financial limitations.

## Data Availability Statement

The datasets presented in this study can be found in online repositories. The names of the repository/repositories and accession number(s) can be found at: https://pdma.punjab.gov.pk/covid19_pdma_reporting_detail, https://www.nih.org.pk/, https://covid19.who.int/.

## Author Contributions

AN conceived and designed the concept and wrote the paper. AN and MB performed the literature review. AN and AA contributed in the data collection. SA and FB helped to provide technical support to collect the data. AN and SI contributed in analysis tools. XS has supervised the work. HZ and SR reviewed the work to improve the outcomes. All authors have read and agreed to the published version of the manuscript.

## Conflict of Interest

The authors declare that the research was conducted in the absence of any commercial or financial relationships that could be construed as a potential conflict of interest.
